# miR-155-5p Silencing Does Not Alter BTLA Molecule Expression in CLL T Cells: Implications for Targeted Immunotherapy

**DOI:** 10.3390/biom15111499

**Published:** 2025-10-24

**Authors:** Agata Kosmaczewska, Lidia Ciszak, Anna Andrzejczak, Anna Tomkiewicz, Anna Partyka, Zofia Rojek-Gajda, Irena Frydecka, Dariusz Wołowiec, Tomasz Wróbel, Agnieszka Bojarska-Junak, Jacek Roliński, Lidia Karabon

**Affiliations:** 1 Laboratory of Immunopathology, Department of Experimental Therapy, Hirszfeld Institute of Immunology and Experimental Therapy, Polish Academy of Sciences, 53-114 Wroclaw, Poland; lidia.ciszak@hirszfeld.pl (L.C.); anna.partyka@hirszfeld.pl (A.P.); irena.frydecka@umw.edu.pl (I.F.); 2 Laboratory of Genetics and Epigenetics of Human Diseases, Department of Experimental Therapy, Hirszfeld Institute of Immunology and Experimental Therapy, Polish Academy of Sciences, 53-114 Wroclaw, Poland; anna.andrzejczak@hirszfeld.pl (A.A.); anna.tomkiewicz@hirszfeld.pl (A.T.); lidia.karabon@hirszfeld.pl (L.K.); 3 Dental Center STOMO Clinic, 53-411 Wroclaw, Poland; zofiarojekgajda@gmail.com; 4 Department and Clinic of Hematology, Blood Neoplasms, and Bone Marrow Transplantation, Wroclaw Medical University, 50-367 Wroclaw, Poland; dariusz.wolowiec@umw.edu.pl (D.W.); tomasz.wrobel@umw.edu.pl (T.W.); 5 Department of Clinical Immunology, Medical University of Lublin, 20-093 Lublin, Poland; agnieszkabojarskajunak@umlub.pl (A.B.-J.); jacek.rolinski@gmail.com (J.R.)

**Keywords:** CLL, miR-155-5p, epigenetic modification, BTLA, T cells, immunotherapy

## Abstract

Given that we have demonstrated that miR-155-5p is increased in CLL PBMCs and that its reduction with inhibitory siRNA partially restores the immune checkpoint BTLA protein level in CLL B cells, risk stratification for using anti-miR-155-based immunotherapy in CLL seems reasonable, particularly with its potential impact on T cells. Therefore, we aimed to assess the role of miR-155-5p in the epigenetic modification of BTLA levels in CLL T cells, especially since we observed that BTLA expression unfavorably promotes increased proliferative activity and IL-4 secretion in T cells, thus suggesting BTLA malfunction in the CLL T cell subset. Transfection of PBMCs with an inhibitor of miR-155-5p (INH) led to about a ten-fold down-regulation of miR-155-5p levels compared to control siRNA (NC) both in CLL patients and healthy individuals (HC), as assessed by RT-qPCR. Additionally, we did not find any significant differences in BTLA protein expression in T cells after silencing miR-155-5p in either examined group. We demonstrated for the first time that immunotherapy approaches based on systemic administration of anti-miR-155-5p therapeutics would be a favorable strategy in CLL, since they do not affect BTLA expression in T cell populations and could benefit CLL patients with impaired BTLA levels on CLL cells.

## 1. Introduction

Chronic lymphocytic leukemia (CLL) is a hematological malignancy characterized by an accumulation of clonal B cells expressing CD19, CD20, and CD23, and CD5 in the blood, bone marrow, lymph nodes, and spleen. It has been demonstrated that leukemic transformation of B cells could be initiated by specific genomic alterations [1]. Furthermore, molecular heterogeneity of CLL was also described, identifying potential genomic biomarkers for prognosis and/or response to therapy in CLL [2]. Among them, pathogenic and prognostic significance could be assigned to epigenetic regulatory events, including microRNAs (miRNAs) [2]. Transcriptional profiling studies identified a variety of differentially expressed miRNAs in CLL [3,4]. Moreover, several miRNAs have been found to associate with prognostic factors, disease-specific karyotype aberrations, and CLL outcome, indicating their prognostic potential in CLL [5,6,7,8,9,10]. miRNAs are short endogenous non-coding RNAs that epigenetically negatively regulate gene expression. They act through binding to the 3′ untranslated region (3′UTR) of the targeted messenger RNA (mRNA) molecules, thus resulting in translation repression or/and mRNA degradation [11,12]. It has been demonstrated that approximately 60% of protein-encoding genes are regulated by different miRNAs that control the activity and function of signaling molecules and cellular processes, such as proliferation, apoptosis, and cell differentiation [13,14]. In fact, miRNAs, through their interaction with targets, have been shown to be involved in cell cycle regulation, where they thereby can act as either oncogenes or tumor suppressors [15]. Deregulated miRNA expression, including miR-15a, miR-16, miR-181b, and the miR-17/92 cluster, were reported in CLL onset and progression [16,17,18,19,20]. An imbalance in miRNAs and their targets may disrupt leukemic cell cycle progression and lead to an increased proliferation rate [11]. miR-155 is a highly conserved proinflammatory and oncogenic miRNA, ubiquitously expressed in activated B and T cells, as well as macrophages [13]. It is encoded by the *MIR155HG* gene located on chromosome 21q21, and is involved in normal B cell differentiation [21,22,23]. miR-155 levels in humans are low in normal lymphoid tissues, but accumulate in malignancies. Recently, miR-155-5p overexpression in PBMCs in CLL patients was reported as a novel molecular biomarker of disease aggressiveness and poor prognosis [19,20,24]. Furthermore, previous research also showed that pre-treatment levels of the miR-155 may have predictive significance in CLL. In this regard, patients with the lower miR-155 expression in plasma samples collected before treatment achieved complete remission or exhibited stabilization of the disease in comparison to all others [21]. These data indicated miR-155 as a useful marker to identify CLL patient responsiveness to therapy and/or to predict clinical outcome [13]. Targets of miR-155-5p include several cytoplasmic and nuclear proteins, including immune checkpoint molecules [11,25].

Patients with CLL progressively develop immunosuppression, dampening anti-leukemic response. It has been reported that CLL progression is associated with increased expression of several immune checkpoints, such as LAG-3, TIGIT, NKG2, and ILT2, as well as functional features of T cell exhaustion, which compromises the anti-tumor immune response [26,27,28,29,30]. Nonetheless, in the available literature, there are inconsistent results concerning immune checkpoint expression (i.e., BTLA) in PBMCs from CLL patients [31,32,33]. BTLA (B- and T-lymphocyte attenuator, also known as CD272) is an immunoglobulin (Ig) superfamily member that acts as an inhibitory immune checkpoint. Structurally, BTLA is a type I transmembrane glycoprotein of approximately 32 kDa, consisting of an extracellular immunoglobulin (Ig) domain, a transmembrane region, and a cytoplasmic tail that contains three highly conserved tyrosine-based motifs: a growth factor receptor-bound protein 2 (Grb2) binding site, an immunoreceptor tyrosine-based inhibitory motif (ITIM), and an immunoreceptor tyrosine-based switch motif (ITSM) [34,35]. The coexistence of both inhibitory (ITIM/ITSM) and activating (Grb2 binding) motifs makes BTLA a unique receptor capable of potential bidirectional signaling [36]. Upon binding to its ligand, the herpesvirus entry mediator (HVEM) BTLA —a member of the TNF receptor superfamily (TNFRSF14)—recruits the phosphatases SHP-1 and SHP-2, primarily signaling through SHP-1, to suppress T cell activation. Primarily, BTLA is expressed on B and T cells (both CD4^+^ and CD8^+^), while also on NK cells, macrophages, and dendritic cells. Its expression often increases when immune cells become chronically stimulated or exhausted, as in cancer or chronic infection. The physiological role of this immune checkpoint is to maintain immune tolerance and regulate the balance between immune activation and suppression in normal physiology. In tumors, similar to PD-1, BTLA dampens antitumor immunity, and high BTLA expression is frequently linked to T cell exhaustion in the tumor microenvironment. The literature data indicate BTLA upregulation in various cancers, including gallbladder cancer, melanoma, lung cancer, DLBCL, RCC, etc., which is often associated with impaired antitumor T cell responses. However, some studies have reported contradictory observations, for instance, BTLA levels were found to be decreased in colorectal cancer tissues in comparison with levels in matched non-carcinoma tissues [37].

In CLL, contradictory results on BTLA expression have been reported, indicating the importance and complexity of CLL immunopathology. In some studies, an augmented expression of BTLA in leukemic B cells and NK cells from CLL patients has been reported [31]. Also, elevated BTLA expression in NK and T cells from CLL was shown to correlate with a shorter time to treatment as well as a diminished anti-tumor response, while treatment with an anti-BTLA blocking antibody restored anti-leukemic activity by promoting in vitro Th1 cytokine production and cytotoxicity [31,32]. In contrast, in our former study, a lower expression of BTLA in leukemic B cells and its association with an increased proliferation of BTLA+ B cells were found, suggesting that impaired BTLA levels might contribute to lowering the threshold for B cell activation and proliferation [33]. Moreover, we also showed that inappropriate BTLA molecule expression in leukemic B cells is epigenetically regulated by an elevated level of miR-155-5p and could be partially restored by the inhibition of miR-155-5p [25]. While immune checkpoint inhibitor-based therapy did not provide benefits in clinical trials in CLL patients [38], some trials, including those with anti-BTLA monoclonal antibodies in hematological malignancies, are still ongoing (NCT04477772); however, no results have been published so far. These contradictory reports clearly show the need to evaluate the implications of therapeutic manipulation of BTLA expression for CLL patients.

Therefore, the aim of the present study was to determine whether potential anti-miR-155 therapy in CLL, which may restore/improve BTLA expression in the B cell population, increasing their activation threshold, could pose a risk in terms of its potential simultaneous effect on the T cell population when administered systemically. What might be the consequences of such an approach for the T cell population, and how might it influence the course of CLL? Addressing this issue is of great clinical importance, since, in our previous two reports, we demonstrated that non-malignant T cells are able to influence CLL outcomes [39,40].

## 2. Materials and Methods

### 2.1. Patients and Controls

In our study, peripheral blood mononuclear cells (PBMCs) of 20 patients with CLL, who had not received any chemotherapy regimen, attending the Clinical Department of Hematology, Cell Therapies and Internal Diseases, Wroclaw Medical University (Wroclaw, Poland) and 15 age- and sex-matched healthy controls, recruited from the Hirszfeld Institute of Immunology and Experimental Therapy, Polish Academy of Sciences (Wroclaw, Poland) and the Lower Silesian Oncology Center (Wroclaw, Poland), were analyzed.

All patients enrolled for screening were evaluated using the generally accepted criteria of CLL diagnosis based on clinical examination, peripheral blood cell count, cell morphology, and immunophenotyping analysis [1,41]. Only patients in good general condition and without indications for anti-leukemic therapy were included in this study. Exclusion criteria included the presence of any other cancer, as well as comorbidities or/and treatment interfering with the immune responses (e.g., corticosteroids or immunomodulatory drugs) in the present or past. The Rai and Binet classification systems were used to determine the stage of CLL.

The same criteria were used for healthy individuals’ enrollment. All persons participating in the study were informed about the purpose and details of the study. The project was approved by the local Bioethical Committee at the Medical University of Wroclaw, Poland (approval no. KB-321/2010), and is in accordance with the Helsinki Declaration of 1975. Before any medical procedure was performed, every participant signed two copies of the written informed consent form and was given one of them.

### 2.2. Peripheral Blood Mononuclear Cell (PBMC) Isolation

Venous blood from CLL patients and healthy subjects was collected in CPTs containing sodium heparin anticoagulant. PBMCs were isolated by density gradient centrifugation using Lymphoflot reagent (Cat. No. #824012, Bio-Rad, Dreieich, Germany) and washed three times in phosphate-buffered saline (PBS) without Ca^+2^ and Mg^+2^. Isolated PBMCs were frozen in a suspension containing 95% fetal calf serum (Cat. No. #CA5-104, CytoGen GmbH, Sinn, Germany) and 5% dimethylsulfoxide (DMSO, Cat. No. #D8418, Merck KGaA, Darmstadt, Germany), and were refrozen directly before further investigation.

### 2.3. Determination of BTLA mRNA Levels in T Cell Subpopulation

The studies presented in this section were performed on 40 CLL patients recruited from Department of Clinical Immunology, Medical University of Lublin (Lublin, Poland), and 17 healthy individuals. BTLA mRNA expression results obtained from a subset of 21/40 patients were in part described previously [33]. Patient characteristics for this part of the study are presented in Supplementary Materials (Table S1).

Cell separation was performed in order to determine the BTLA mRNA levels in the different subpopulations. Briefly, PBMCs previously frozen were thawed and first subjected to positive selection of T cells (CD3 positive cells), according to the manufacturer’s instructions, using the Human CD3 Selection Cocktail (Cat. No. #18051, StemCell Technologies, Vancouver, BC, Canada), and, subsequently, negative selection of B cells in the remaining material was performed using the Human B cell enrichment kit without CD43 depletion (Cat. No. #19154, StemCell Technologies, Vancouver, BC, Canada) for patients or the Human B cell enrichment kit (Cat. No. #17954, StemCell Technologies, Vancouver, BC, Canada) for controls.

RNA isolation was performed using the Chomczynski method from 1 million cells for each sample [42]. Next, 500 ng of total RNA was reverse-transcribed using the iScript cDNA Synthesis Kit (Cat. No. 1708891, Bio-Rad, Berkeley, CA, USA). Human BTLA mRNA levels were determined using Applied Biosystems assays (Hs00699198_m1), while *β2microglobulin* (*β2M*) was applied as the reference gene using Pre-developed TaqMan Assay Reagents Human β2M). The efficiency of BTLA and *β2M* mRNA expression was 80.3 and 81.4, respectively. All of the samples were assayed in duplicate on the 7300 Real Time PCR System (Applied Biosystems). The results were calculated according to the Δ*C_t_* method [43].

### 2.4. MicroRNA Studies

The studies presented here have been partially described previously [26].

#### 2.4.1. In Silico Analysis

To establish any potential interaction of miR-155-5p with the BTLA gene, we applied three different bioinformatic tools: DIANA (http://diana.imis.athena-innovation.gr/DianaTools/index.php?r=microT_CDS/index accessed on 12 November 2021), TARGET SCAN (http://www.targetscan.org/vert_80/), and mirdip (http://ophid.utoronto.ca/mirDIP/index.jsp#r accessed on 12 November 2021).

#### 2.4.2. Luciferase Test

Plasmid sequences were designed based on in silico prediction analysis. Wild-type (WT) or mutated (MUT) miRNA binding sites located in the 3′ untranslated region (3′UTR) of the BTLA gene were cloned into the pGL4.13[luc2/SV40] vector (Cat. no. E668A, Promega, Madison, WI, USA) and propagated in TOP10 Competent Cells (Invitrogen, Thermo Fisher Scientific Inc., Waltham, MA, USA). Plasmids were purified using the PureYield™ Plasmid Miniprep System (Cat. no. A1223, Promega, Madison, WI, USA). All constructs were verified by restriction enzyme digestion and/or PCR and sequencing to confirm the presence and accuracy of the inserted sequences. WT and MUT oligonucleotides were synthesized using Genomed (Warsaw, Poland). Specifically, two types of WT and MUT constructs were prepared: one containing the full-length BTLA 3′UTR and another containing a ~200 bp fragment encompassing the predicted miR-155-5p binding site. For the MUT constructs, six-nucleotide substitutions were introduced within the miRNA binding site to disrupt the predicted interaction between miR-155-5p and the 3′UTR. In total, six constructs were generated for the dual-luciferase assay: pGL4.13 (Firefly luciferase), pGL4.74 (Renilla luciferase), pGL4.13_3′UTR_BTLA (full-length 3′UTR), pGL4.13_3′UTR_BTLA_mutMRE155 (full-length 3′UTR with mutated MRE-155-5p), pGL4.13_short3′UTR_BTLA (~200 bp fragment with MRE-155-5p), and pGL4.13_short3′UTR_BTLA_mutMRE155 (~200 bp fragment with mutated MRE-155-5p).

Validation of the miRNA–3′UTR interaction was performed in HEK 293 cells. Briefly, HEK 293 cells were co-transfected with WT or MUT constructs together with either a miR-155-5p mimic or negative control (NC) using Lipofectamine 2000 transfection reagent (Cat. no. 11668027, Invitrogen, Thermo Fisher Scientific Inc., Waltham, MA, USA). After 24 h, luciferase activities were measured using the Dual-Glo^®^ Luciferase Assay System (Cat. no. E2920, Promega, Madison, WI, USA) on a GloMax^®^-Multi Detection System (Cat. No. E9032, Promega, Madison, WI, USA). Five independent experiments were performed in triplicate. For each experiment, the ratio of Firefly to Renilla luciferase activity was calculated to normalize for transfection efficiency. Dual-luciferase activities were normalized to the mock control, which was set to 1. For statistical comparison of luciferase activity between WT and MUT constructs, a two-tailed Mann–Whitney U test was used. Results are presented as mean ± standard deviation (SD). Statistical significance was defined as *p* < 0.05. Analyses were performed using GraphPad Prism version 10 (GraphPad Software, San Diego, CA, USA).

#### 2.4.3. miR-155-5p Inhibition

As described previously [25], isolated PBMCs from 20 CLL patients and 15 healthy individuals (patient characteristics are presented in Table 1) were suspended in basic OPTIMEM medium (Cat. no. #11058021, Gibco, Paisley, UK), and 1 × 10^6^ cells per well were seeded into 24-well plates and kept in a humidified 5% CO_2_ incubator at 37 °C for 24 h. Then the cells were transfected with 10 pmol miR-155-5p inhibitor (INH) (Cat. no. #4464084, Invitrogen, Thermo Fisher Scientific Inc., Waltham, MA, USA) or negative control (NC) (Cat. no. #4464076, Invitrogen, Thermo Fisher Scientific Inc., Waltham, MA, USA) using Lipofectamine RNAiMAX Transfection Reagent (Cat. no. #13778030, Invitrogen, Thermo Fisher Scientific Inc., Waltham, MA, USA) according to the manufacturer’s protocol. For better transfection efficacy, the plates were centrifuged for 30 min at 1000 rpm and 37 °C to place the suspended cells at the bottom of well [44]. Cells were incubated for the next 24 h, and then harvested and used for analysis.

#### 2.4.4. Determination of miR-155-5p Expression

For MicroRNA isolation from PBMCs, the miRNA Mini Kit (Cat. No. #SY391210, Syngen Biotech, Wroclaw, Poland) was used according to the manufacturer’s instructions. The cDNA templates were prepared from miRNA using the TaqMan Advanced miRNA cDNA Synthesis Kit (Cat. no. #A28007, Applied Biosystems, Van Allen Way, Carlsbad, CA, USA). RT-qPCR reactions were performed using the TaqMan Universal PCR Master Mix, no AmpErase UNG (Cat. no. #4324018, Applied Biosystems, Warrington, UK) with the miR-155-5p TaqMan Advanced MicroRNA Assay (Cat. no. #A25576, 477927_mir, Applied Biosystems, Pleasanton, CA, USA). The miR-361-5p (Cat. no. #A25576, 478056_mir, Applied Biosystems, Pleasanton, CA, USA) and the miR-186-5p (Cat. No. #A25576, 477940_mir, Applied Biosystems, Pleasanton, CA, USA) were used as controls. The experiment was carried out in duplicate. Quantitative miRNA expression data were acquired using the ViiA7 Real-Time PCR system (Applied Biosystems). The relative miR-155-5p expression levels (relative expression units—RU) were determined using the comparative Ct (2−ΔΔCt) method, using median expression in HC as the calibrator.

### 2.5. Determination of BTLA mRNA Expression in PBMCs

Total RNA was isolated from PBMCs during miR isolation using the miRNA Mini Kit (Cat. no. #SY391210, Syngen Biotech, Wroclaw, Poland) according to the manufacturer’s instructions. The cDNA templates were prepared from total RNA using the High-Capacity cDNA Reverse Transcription Kit (Cat. no. #4368814, Applied Biosystems, Vilnius, Lithuania). RT-qPCR reactions were performed on the ViiA7 Real-Time PCR system (Applied Biosystems) using TaqMan Universal PCR Master Mix, no AmpErase UNG (Cat. No. #4324018, Applied Biosystems, Warrington, UK), with the BTLA TaqMan Gene Expression Assay (Cat. no. #4331182, Hs00699198_m1, Applied Biosystems, Pleasanton, CA, USA). ACTB (Cat. no. #4331182, Hs03023943_g1, Applied Biosystems, Pleasanton, CA, USA) and GAPDH (Cat. no. #4331182, Hs02786624_g1, Applied Biosystems, Pleasanton, CA, USA) were used as housekeeping genes. The experiment was carried out in duplicate. The relative BTLA mRNA expression levels (RUs) were determined using the comparative Ct (2−ΔΔCt) method, and median expression in HC was used as the calibrator [25,33].

### 2.6. Determination of BTLA Protein Expression in T Cell Subpopulation

Surface and intracellular expression of the BTLA molecule in peripheral blood CD3+ cells of CLL patients and healthy controls was determined by double-immunostaining with flow cytometry using the following monoclonal antibodies: CD3-PerCP (Cat. no. #552851, BD Pharmingen, BD Biosciences, San Diego, CA, USA), BTLA-PE (Cat. no. #558485, BD Pharmingen, BD Biosciences, San Diego, CA, USA), and the appropriate isotype controls. Surface and intracellular expression of the BTLA protein in CD3+ cells was studied according to standard protocols and the instructions described in detail by Karabon et al. [33].

To assess the surface expression of the BTLA protein on CD3+ cells after the culture with miR-155-5p inhibitor (INH) or negative control (NC) (as described above), the cells were washed and aliquoted into tubes for further surface staining of CD3 and BTLA with monoclonal antibodies (MoAbs) conjugated with fluorochromes according to standard protocols. Directly after immunostaining, the cells were washed and analyzed by flow cytometry using a FACScan cytometer (Becton Dickinson, BD Biosciences, San Diego, CA, USA) equipped with CellQuest software 2.0 for data analysis (Becton Dickinson, BD Biosciences, San Diego, CA, USA). At least 50,000 events per sample were analyzed in each experiment. The percentages of BTLA+ cells and mean fluorescence intensity (MFI) value expressed in arbitrary units (AUs) were determined in a subset of CD3+ lymphocytes.

### 2.7. Assessment of T Cell Capacity for IL-4 Secretion and Proliferation

Expression of Ki67 and IL-4 proteins in peripheral blood BTLA-positive and BTLA-negative CD3+ cells of CLL patients and healthy controls was determined by triple-immunostaining with flow cytometry using the following monoclonal antibodies: CD3-PerCP (Cat. no. #552851, BD Pharmingen, BD Biosciences, San Diego, CA, USA), BTLA-PE (Cat. no. #558485, BD Pharmingen, BD Biosciences, San Diego, CA, USA), IL-4-FITC (Cat. no. #554484, BD Pharmingen, BD Biosciences, San Diego, CA, USA) or Ki67-FITC (Cat. no. #556026, BD Biosciences, San Diego, CA, USA), and the appropriate isotype controls.

For induction of the intracellular expression of IL-4 cytokine and proliferation marker Ki67, the thawed PBMCs were incubated with polyclonal stimulators in short-term cultures. Briefly, the cells were suspended at 1 × 10^6^ PBMCs/mL in RPMI 1640 medium (Cat. no. #10432512, Gibco, Paisley, UK) supplemented with 10% fetal calf serum (Cat. no. #CA5-104, CytoGen GmbH, Sinn, Germany), 2 mmol/L L-glutamine (Cat. No. #J16285.A1, Gibco, Paisley, UK), 50 µg/mL gentamicin (Cat. no. #15750060, Gibco, Paisley, UK), and cultured with 25 ng/mL phorbol 12-myristate 23-acetate (PMA, Cat. no. #P8139, Sigma-Aldrich, Merck KGaA, Darmstadt, Germany) and 1 µg/mL ionomycin (Ion, Cat. no. #I0634, Sigma-Aldrich, Merck KGaA, Darmstadt, Germany) in the presence of 10 µg/mL brefeldin A (BFA, protein transport inhibitor, Cat. no. #B6542, Sigma-Aldrich, Merck KGaA, Darmstadt, Germany) for 4 h at 37 °C in a humidified atmosphere containing 5% CO_2_. Next, the cultured cells were stained with anti-CD3 and anti-BTLA MoAbs, and were then fixed and permeabilized with the Fixation/Permeabilization Buffer Set (Cat. no. #88-8824-00, eBioscience, San Diego, CA, USA) according to the manufacturer’s instructions. The efficacy of permeabilization was determined by the uptake of trypan blue. Following washing, the cells were incubated for 30 min at 4 °C with anti-IL-4 or anti-Ki67 MoAbs conjugated with fluorochromes. Isotype-matched control antibodies were used to confirm expression specificity.

Directly after immunostaining, the cells were washed and analyzed by flow cytometry using a FACScan cytometer equipped with CellQuest software (Becton Dickinson, BD Biosciences, San Diego, CA, USA). We analyzed the proportions of IL-4+ or Ki67+ cells within both CD3+BTLA+ and CD3+BTLA− cells. At least 50,000 events per sample were analyzed in each experiment. The gating strategy is presented in Figure S1 in Supplementary Materials.

### 2.8. Statistical Analysis

Statistical analyses of the clinical data and laboratory findings were conducted using the Statistica 10.0 package (Tibco Software Inc., Palo Alto, CA, USA) and GraphPad Prism 8.01 (GraphPad Software, San Diego, CA, USA). For clinical parameters of CLL patients, the mean values and standard deviation (SD) are presented. Median values and interquartile ranges were calculated additionally for all other variables. All collected data were examined for normal distribution using the Shapiro–Wilk test. For normally distributed data, the comparisons between the studied groups were performed using Student’s *t*-test for independent samples. In case of a non-normal distribution, the Mann–Whitney U test for comparison between groups was used. To test the effects of stimulation, as well as the effects of miR-155-5p inhibition, Student’s *t*-test for dependent samples and the non-parametric Wilcoxon signed-rank test were applied. In all analyses, differences were considered significant when *p* ≤ 0.05.

## 3. Results

### 3.1. Patient and Control Characteristics for the miR-155-5p Study

A total of 20 patients with previously untreated CLL and 15 healthy controls were recruited into the study between March 2003 and October 2016. Patients were enrolled from the Department and Clinic of Hematology, Blood Neoplasms, and Bone Marrow Transplantation, Wroclaw Medical University (Wroclaw, Poland). All of the patients fulfilled the above-mentioned inclusion and exclusion criteria. Their clinical and laboratory characteristics are summarized in Table 1.

### 3.2. BTLA mRNA Expression in T Cells

**Figure 1:**
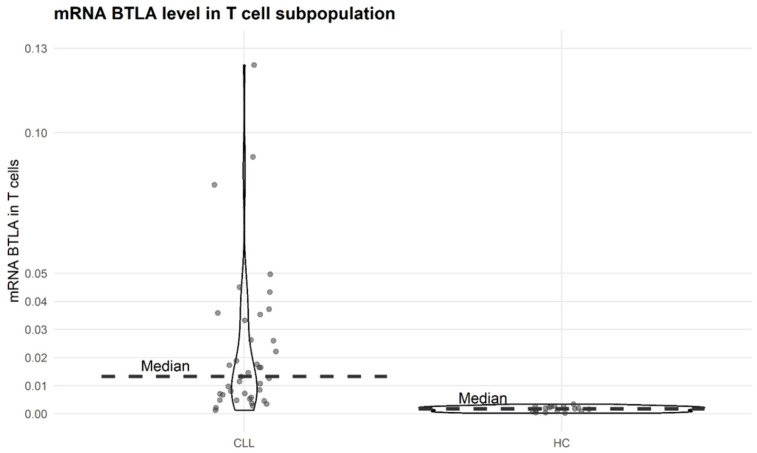
BTLA mRNA expression levels in CLL patients (*n* = 40) and healthy controls (HC) (*n* = 17) in T cell subpopulation (CD3+), Mann–Whitney U test. *p* = 2.438 × 10^−8^.

In peripheral blood, the median BTLA mRNA expression in T cells was 7.75 times higher than that in controls (2-delta CT was 0.0132 vs. 0.0017, *p* = 2.438 × 10^−8^). As can be clearly seen, the range of results in CLL patients is wider than in controls (0.012–0.12415 vs. 0.0003–0.0034, Figure 1).

### 3.3. BTLA Molecule Expression in T Cells

**Figure 2:**
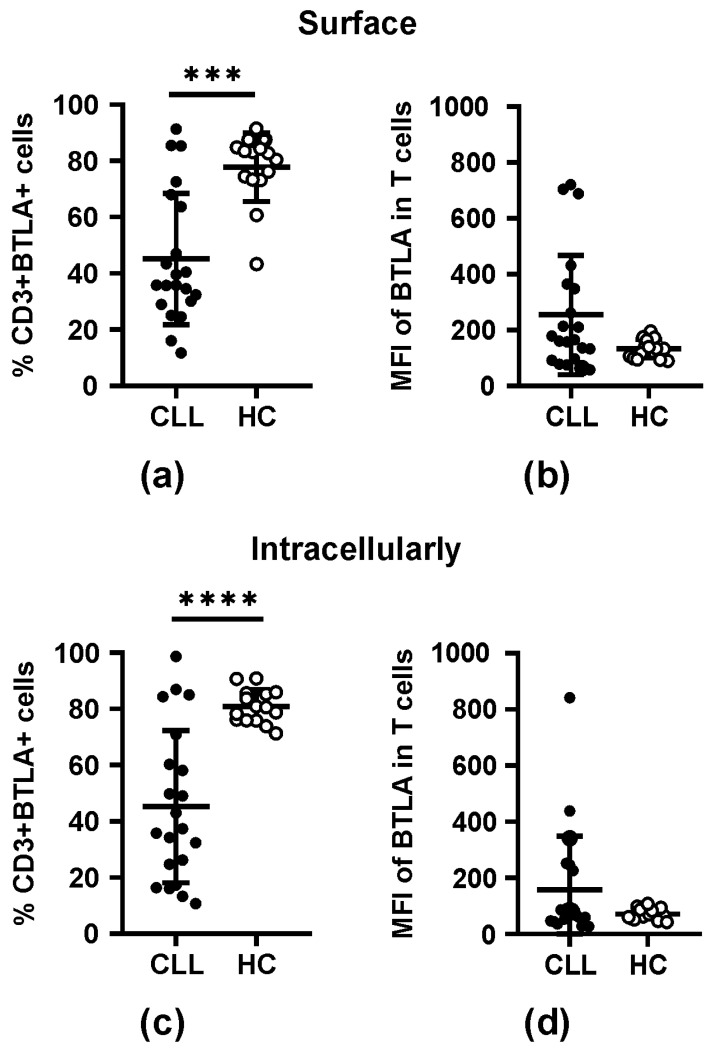
Comparison of BTLA expression in peripheral blood T cells between patients with CLL and healthy controls. (**a**,**c**) Surface (**a**) and intracellular (**c**) BTLA expression in T (CD3+) cell subpopulations from CLL patients and healthy controls (HC). (**b**,**d**) The mean fluorescence intensity (MFI) of BTLA molecules on the surface (**b**) and intracellularly (**d**) in T cells from CLL patients and healthy controls. The horizontal lines represent the mean and SD values. Differences between the groups studied were evaluated using Student’s t-test for independent samples or the Mann–Whitney U test. (***) and (****) signify a statistically significant differences of *p* < 0.001 and *p* < 0.0001, respectively.

To find out whether surface BTLA expression in CLL patients might result from affected recycling to the cell membrane, we performed intracellular staining for cytoplasmic expression of BTLA molecules. Given that we found a similar pattern of BTLA expression both at the cell surface and in the cytoplasmic compartment (Figure 2), we could exclude disturbed intracellular BTLA trafficking as the cause of surface BTLA expression changes in CLL T cells, suggesting instead alterations in the BTLA gene activity or epigenetic regulation.

In line with the increased transcriptional activity of the BTLA gene in CLL PBMCs, we observed, at the protein level, higher amounts of BTLA molecules either on the T cell surface or intracellularly in CLL patients compared to HC. In line, as shown in Figure 2b,d, on average, we detected about a two-fold increase in BTLA protein levels in CLL T cells; however, the differences compared to BTLA expression in HC T cells were not statistically significant, most probably due to large differences between the results obtained for individual CLL cases (*p* = NS). In detail, surface BTLA MFI values ranged from 57.9 to 703.9, with a mean ± SE of 254.2 ± 213.9 for CLL patients. In HC, surface BTLA MFI varied between 89.8 and 195.7, with a mean ± SE of 133.2 ± 33.1 (Figure 2b). Likewise, for intracellular BTLA protein levels, the mean values were as follows: 156.6 ± 192.8 (ranged from 26.8 to 841.7) for CLL vs. 71.5 ± 19.2 (ranged from 43.1 to 107.6) for HC.

Qualitative analysis of BTLA protein expression revealed a significantly lower fraction of T cells co-expressing BTLA on the cell surface and intracellularly in the CLL cohort in comparison to HC (Figure 2a,c). The differences between corresponding mean ± SE values in CLL and HC were as follows: 45.1 ± 23.3% vs. 77.7 ± 12.2% (*p* = 0.0002) and 45.3 ± 27.1 vs. 80.9 ± 5.9% (*p* = 0.00002), respectively.

### 3.4. The Impact of BTLA Molecule Expression on T Cell Effector Functions

Given that aberrant BTLA expression in CLL has been demonstrated, as we previously reported in the B cell population [25,33], we aimed to verify whether BTLA molecule expression and its eventual modification at the epigenetic level might affect T cell effector functions. To address this issue, we performed functional analyses to assess the capacity of CLL T cells for proliferation and IL-4 secretion in relation to BTLA expression, and the results obtained were compared to the values from HC.

**Figure 3:**
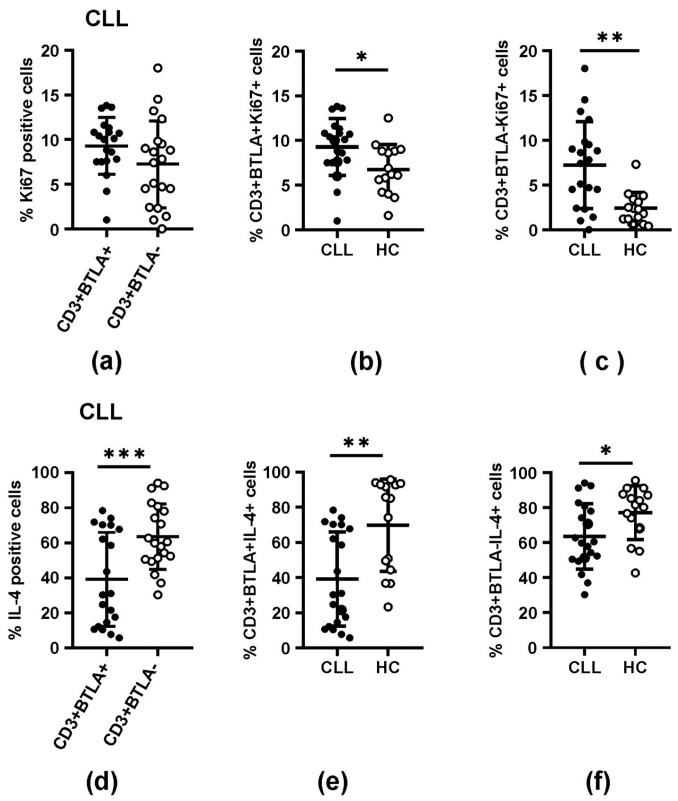
Expression of Ki67 and IL-4 proteins in BTLA+ and BTLA− T cell populations (%) in CLL patients and healthy controls. (**a**,**d**) Comparison of Ki67 (**a**) and IL-4 (**d**) expression between BTLA+ and BTLA− T cell subsets in CLL patients. (**b**,**c**,**e**,**f**) Comparison of Ki67 (**b**,**c**) and IL-4 (**e**,**f**) expression in BTLA+ and BTLA-T subsets between CLL patients and healthy controls. The horizontal lines represent the mean and SD values. Differences between the groups studied were evaluated using Student’s *t*-test for independent samples or the Mann–Whitney U test. (*), (**), and (***) denote statistically significant differences of *p* < 0.05, *p* < 0.01, *p* < 0.001, respectively.

Comparative analysis between CLL Ki67+ BTLA-positive and Ki67+ BTLA-negative T cell compartments showed no significant differences (Figure 3a), while in HC, we observed a higher proportion of Ki67+ BTLA-positive T cells over Ki67+ BTLA-negative T cells (*p* = 0.0001) (Table 2). In addition, a significant increase was observed in the proportions of Ki67+ BTLA-positive and Ki67+ BTLA-negative T cells in CLL patients compared to the corresponding cells in HC (*p* = 0.02 and *p* = 0.0015, respectively) (Table 2, Figure 3b,c).

Unlike proliferative activity, we observed, in CLL patients, a significant decrease in the IL-4+ BTLA-positive T cell compartment compared to the IL-4+ BTLA-negative T cell fraction (*p* = 0.008) (Table 2, Figure 3d). In HC, no difference between IL-4+ BTLA-positive and IL-4+ BTLA-negative T cell frequencies was found (Table 2). Moreover, both IL-4+ BTLA-positive and IL-4+ BTLA-negative fractions from CLL patients were found to be significantly decreased in comparison to corresponding T cells in HC (*p* = 0.0016 and *p* = 0.0286, respectively) (Table 2, Figure 3e,f).

**Figure 4:**
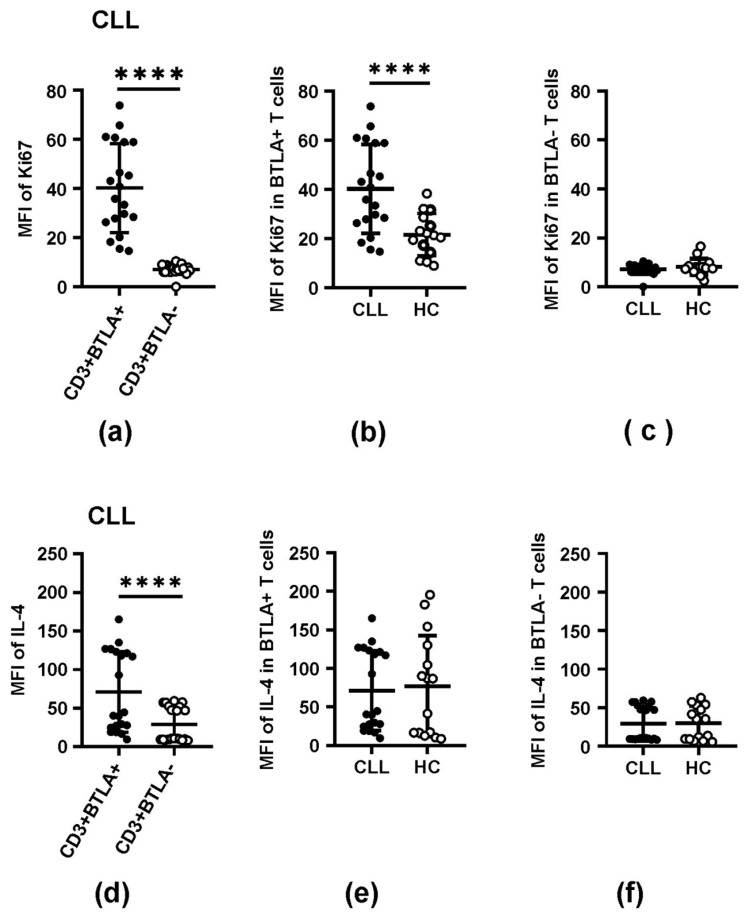
Expression of Ki67 and IL-4 proteins (MFI) in BTLA+ and BTLA− T cell populations in CLL patients and healthy controls. (**a**,**d**) Comparison of the expression levels of Ki67 (**a**) and IL-4 (**d**) proteins between BTLA+ and BTLA− T cell subsets in CLL patients. (**b**,**c**,**e**,**f**) Comparison of the expression levels of Ki67 (**b**,**c**) and IL-4 (**e**,**f**) proteins in BTLA+ and BTLA− T subsets between CLL patients and healthy controls. The horizontal lines represent the mean and SD values. Differences between the groups studied were evaluated using Student’s t-test for independent samples or the Mann–Whitney U test. (****) denote statistically significant difference *p* < 0.0001.

Quantitative analysis of Ki67 and IL-4 expression confirmed their higher expression in BTLA-positive T cells compared to the values seen in BTLA-negative T cells in all participants studied (*p* ≤ 0.034) (Table 3, Figure 4). When comparing CLL and HC groups, we observed statistically similar levels of Ki67 and IL-4 both in BTLA-positive and BTLA-negative T cell subsets, except for Ki67 MFI, detected in patients’ BTLA-positive T cells, which was significantly increased compared to that seen in the corresponding HC population (*p* = 0.0008) (Table 3, Figure 4).

### 3.5. miR-155-5p Binding to the 3′UTR of the BTLA Gene

**Figure 5:**
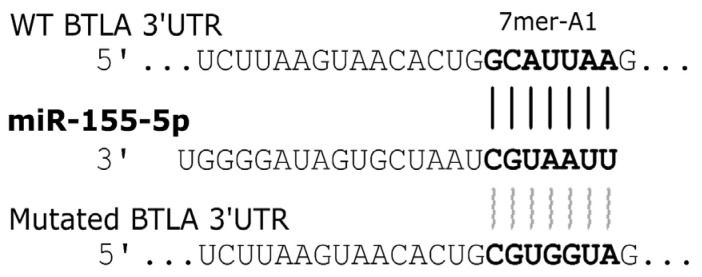
Computational predictions indicated that the 3′UTR region of human BTLA mRNA serves as a potential target for miR-155-5p. Bioinformatic examination of the BTLA gene sequence identified a canonical 7mer-A1 binding motif within its 3′UTR. This motif corresponds to a perfect match with nucleotides 1524–1530 of the mature miR-155-5p, followed by an adenine residue. The predicted binding site sequence is 5′-GCAUUAA-3′. The alignment of miR-155-5p with both the wild-type (WT) and mutant BTLA 3′UTR sequences in the luciferase constructs is presented.

**Figure 6:**
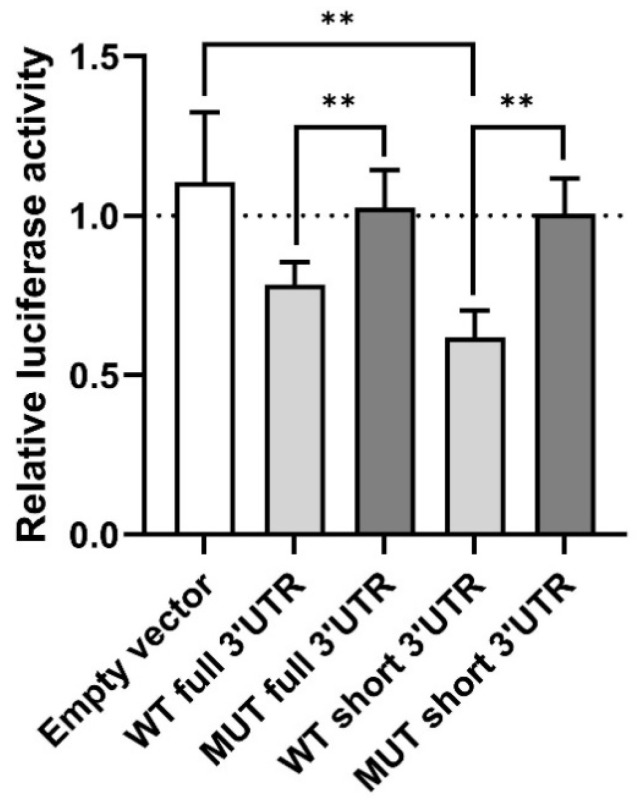
Interaction between miR-155-5p and the BTLA 3′UTR demonstrated by dual-luciferase reporter assay in HEK 293 cells. *WT* indicates the wild-type BTLA 3′UTR; *MUT* indicates the BTLA 3′UTR containing a mutation within the predicted miR-155-5p binding site. Data are presented as the relative change in luciferase activity in comparision to mutated fragment of 3′UTR (no miR155-5-p binding) (mean ± SD from five independent experiments, each performed in triplicate). ** *p* < 0.01. Dot line means 1 (no changes).

Analysis using three different bioinformatic tools revealed a putative miR-155-5p binding site within the BTLA gene sequence, characterized by a canonical 7mer-A1 motif located in the 3′UTR at positions 1524–1530 (5′-GCAUUAA-3′) (Figure 5). To disrupt this predicted interaction, the binding site was mutated to 5′-CGUGGUA-3′. Functional validation using a dual-luciferase reporter assay demonstrated that co-transfection of HEK 293 cells with WT constructs and the miR-155-5p mimic significantly repressed luciferase activity compared to controls. Specifically, a ~20% reduction in relative luciferase activity was observed for the construct containing the full-length BTLA 3′UTR (*p* = 0.008), and a ~40% reduction was observed for the construct containing the short 3′UTR fragment (*p* = 0.008). In contrast, co-transfection of the miR-155-5p mimic with the empty vector or constructs containing the mutated MRE-155-5p showed no significant changes in luciferase activity (Figure 6). These results confirm that miR-155-5p directly targets the BTLA 3′UTR and suggest that the functional miR-155-5p MRE is located at positions 1524–1530 of the BTLA 3′UTR.

### 3.6. The Effect of miR-155-5p Inhibition on BTLA Protein Levels in T Cells

Since BTLA protein expression on CLL T cells does not fully correspond with that observed at the mRNA level, we aimed to assess the possible role of miR-155-5p in the epigenetic modification of BTLA expression in CLL T cells in order to detail its role in CLL pathogenesis. This hypothesis is supported by our recent finding that miR-155-5p is increased in CLL PBMCs and that its reduction with inhibitory siRNA partially restores the BTLA surface protein levels in CLL patient B cells [25]. Therefore, in the present study, we decided to determine the functionality of miR-155-5p in T cells from the same CLL patient cohort.

**Figure 7:**
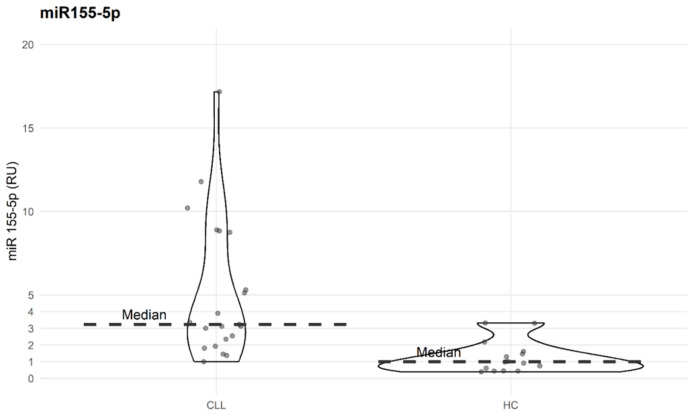
Expression level of miR155-5p in PBMC of CLL patients (*n* = 20) and HC (*n* = 15). Median upregulation for CLL patients is 3.274-fold higher than the median for HC (used as calibrator in Δ ΔCT method), Mann–Whitney U test, *p* = 2.573 × 10^−5^).

As we reported previously [25], miR-155-5p is overexpressed in CLL patients compared to HC. In our study, we compared the expression levels of miR-155-5p in CLL patients to healthy individuals, taking the median for the latter as 1 (ranging from 0.396 to 3.349 RU). In CLL PBMCs, miR-155-5p expression level ranged from 1.011 to 17.370 RU, with a median of 3.274 RU. The difference between the groups was statistically significant (Figure 7).

**Figure 8:**
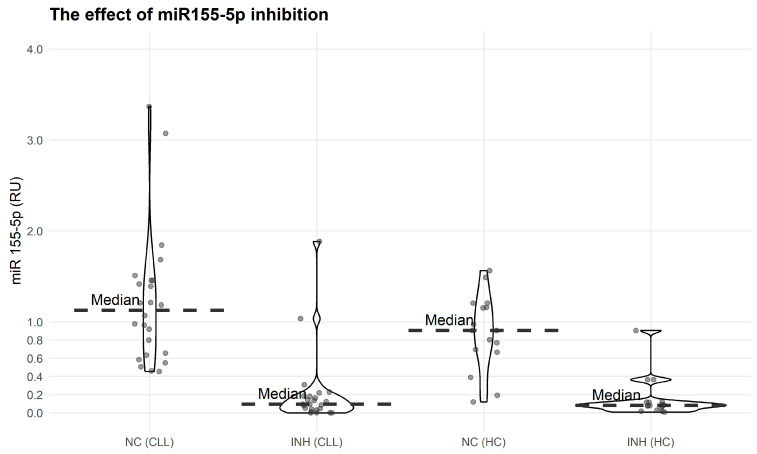
The effect of miR-155-5p silencing with use of a miR-155-5p inhibitor (INH) or control siRNA (NC) in CLL (20) patients and HC (15). RU was calculated using expression in untreated PBMCs as a calibrator. Mann–Whitney U test: NC (CLL) vs. INH (CLL)—*p* = 1.539 × 10^−7^; NC (HC) vs. INH (HC)—*p* = 8.594 × 10^−6^ (median: NC (CLL) = 1.12723; INH (CLL) = 0.09576); range (CLL): NC: 0.453953–3.6764, INH: 0.11995–1.56542; median: NC (HC) = 0.90579; INH (HC) = 0.08181; range (HC): NC: 0.00030–1.88538, INH: 0.00897–0.90513.

Subsequently, we silenced miR-155-5p in PBMCs from CLL patients and HC with the use of a miR-155-5p inhibitor (INH). As shown in Figure 8, in both groups studied (CLL and HC), transfection of PBMCs with the inhibitor of miR-155-5p (INH) led to an approximately 10-fold down-regulation of miR-155-5p expression level (INH vs. NC median RU 0.0888, *p* = 0.0023 for CLL and INH vs. NC median RU = 0.082, *p* = 0.045 for HC).

**Figure 9:**
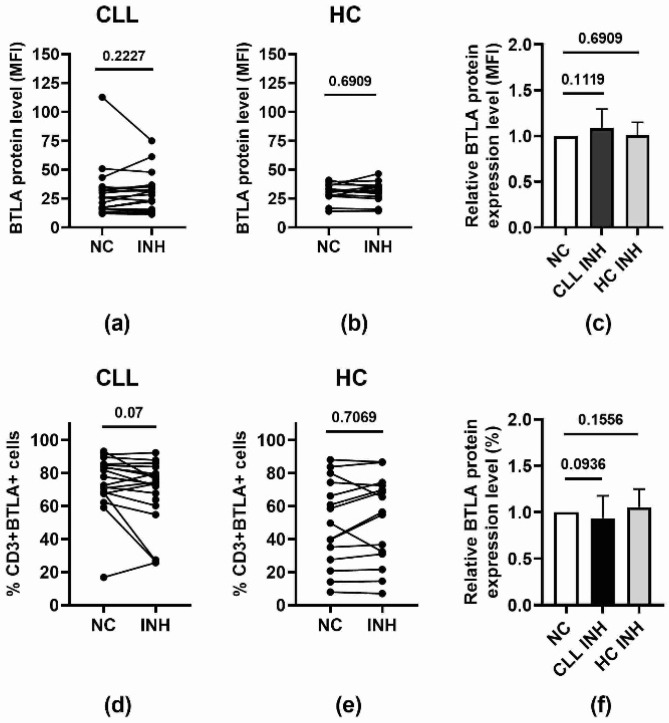
Effect of miR-155-5p inhibition on BTLA protein levels in BTLA+ T cells. (**a**,**b**) Comparison of the level of BTLA protein expression on CD3+ cells in CLL patients (*n* = 19) (**a**) and healthy controls (HC) (*n* = 15) (**b**) transfected with miR-155-5p NC or INH. (**c**) Effect of miR-155-5p inhibition on BTLA protein levels (MFI) on T cells in both CLL patients and healthy controls. Relative protein expression level (R) was calculated for every patient as a fold change in MFI level after treatment with miR-155-5p INH compared to miR-155-5p NC (R = MFI of INH/MFI of NC). The graphs represent the mean and SD of the results of all individuals in each group. (**d**,**e**) The frequency of BTLA-positive CD3+ cells in CLL patients (*n* = 19) (**d**) and healthy controls (HC) (*n* = 15) (**e**) transfected with miR-155-5p NC or INH. (**f**) Effect of miR-155-5p inhibition on BTLA protein expression on T cells in both CLL patients and healthy controls. Relative protein expression level (%) was calculated for every patient as a fold change in frequency level after treatment with miR-155-5p INH compared to miR-155-5p NC (R = frequency of INH/frequency of NC). Differences between the groups studied were evaluated using Student’s *t*-test for dependent samples and the non-parametric Wilcoxon signed-rank test.

As shown in Figure 9, considering the influence of miR-155-5p deficiency on BTLA expression in T cells (Figure 9a–c), we compared the mean fluorescence intensity (MFI) of BTLA protein in CD3+BTLA+ cells between PBMCs transfected with NC and INH, and we did not find any significant differences in either examined group. Accordingly, we did not find any marked influence of miR-155-5p inhibition on the BTLA+ fraction in the T cell population in all individuals studied (Figure 9d–f). Similarly, the pattern of MFI differences in CLL patients and HC was observed to be the same (Figure 9a,b,d,e); however, in the latter group, the distribution of the transfected CD3+BTLA+ cells varied more between individual cases (Figure 9e). From these results, it seems that inhibition of miR-155-5p in CLL T cells does not affect BTLA protein expression.

## 4. Discussion

miR-155-5p has been identified as one of the most up-regulated miRNAs in numerous solid and hematologic malignancies, such as diffuse large B cell lymphoma, acute myeloid leukemia, Hodgkin lymphoma, certain types of Burkitt lymphomas, and CLL [45,46,47,48,49,50,51,52,53]. Additionally, transgenic mice overexpressing miR-155 in B cells clearly demonstrated a decisive role of miR-155 in the molecular mechanisms of B cell development and lymphomagenesis [49]. These findings demonstrate that miR-155 does have a leukemic potential via its direct involvement in the initiation and progression of B cell malignancies. The mechanism underlying the leukemic potential of miR-155p has been linked to cell cycle deregulation in leukemic cells [11]. Targets of miR-155-5p include several cytoplasmic and nuclear proteins that interact with cell cycle regulators (i.e., cyclins/cyclin-dependent kinases), checkpoint proteins, tumor-suppressor molecules, and/or transcription factors regulating numerous genes involved in CLL proliferation, migration, angiogenesis, energy metabolism, and apoptosis [11,15,54,55]. Although over one thousand miR-155 targets have been experimentally identified in all cell types [56], only a few of them were found to be associated with CLL pathogenesis. Taking into account that miR-155-5p overexpression has tumor-promoting activity, being one of the most commonly up-regulated miRNAs in the majority of tumors (rev. in [16]), miR-155 could serve as a therapeutic target for the treatment of cancer. In fact, several studies have demonstrated successful inhibition of miR-155 activity using nanoparticle-based therapy or synthetic inhibitors in B cell malignancies, including CLL [11,57,58].

Recent data confirm that a disrupted balance between miR-155 and its targets could represent one of the molecular mechanisms underlying the increased proliferation rate of leukemic B cells due to cell cycle deregulation [11]. In fact, miR-155 has been implicated in cell cycle control by the inhibition of tumor-suppressor molecules, such as TP53 and cyclin/cyclin-dependent kinases (CDK) [15,18,19,20,59]. In this regard, miR-155 overexpression was shown to affect cell proliferation and cell cycle kinetics by increasing the S and G2 phase population in the breast cancer cell line MCF-7 [59]. Conversely, miR-155 deficiency led to cell cycle arrest in G1 phase and induced apoptosis of these cells as a result of *TP53* mRNA overexpression [59]. In line with these findings, negative relationships between elevated miR-155 levels and the deficiency in cell regulators, such as PU.1, TP53INP1, p21 (CDKN1A), and cyclin D1 (CCND1), have also been reported in CLL [11]. Those miR-155 targets are considered to be associated with B cell development, cell cycle, differentiation of B cells, and CLL pathogenesis [11,16]. The authors used CRISPR/Cas9 gene-editing technology to delete a short sequence encoding the mature miR-155-5p within the *MIR155HG* gene to show, for the first time, that deficiency of miR-155-5p in leukemic B cells (CLL line MEC-1) results in cell cycle arrest and leads to a partial restoration of healthy B cell phenotypes [11].

Given our recent findings, together with other reports demonstrating an increase in miR-155-5p expression in CLL patient samples [13,16,25], risk stratification for using anti-miR-155-based immunotherapy in CLL seems reasonable with regard to its potential impact on T cells alongside B cells. In fact, recent studies from our group reported the involvement of T cell compartment in CLL pathogenesis and clinical outcome, as we demonstrated that the increased turnover of non-malignant T cells could determine CLL progression [39,40]. Our present study clearly demonstrates that CLL T cells, irrespective of BTLA molecule expression, have significantly increased proliferative activity in comparison to corresponding healthy cells. Since BTLA is a suppressor molecule essential for preventing the initiation of immune responses [60,61,62], our present observation on the prevalence of BTLA-negative cells within CLL T cells would explain their augmented proliferative response, but not that of BTLA-positive ones. However, this functional characteristic of CLL T cells appears to be at least partly dependent on BTLA expression, since quantitative analysis of Ki67 expression within CLL T cells displayed an even higher proliferative potential of BTLA-positive T cells compared to BTLA-negative ones. Regarding IL-4 secretion, we observed that both BTLA-positive and BTLA-negative subsets exhibited a significantly lower population of CLL T cells secreting IL-4 in comparison to relevant subsets of healthy T cells. This may support the above-mentioned suggestion concerning the influence of disease-related factors on T cell dysfunction.

While down-regulation in the fraction of IL-4-positive within BTLA-positive CLL T cells may suggest sustained T cell inhibition mediated by BTLA, substantially elevated levels of IL-4 in these cells strengthen the assumption regarding BTLA dysfunction in CLL T cells manifested by the ineffective inhibition of T cell proliferative capacity and IL-4 cytokine secretion. Moreover, given that IL-4 is involved in the survival and proliferation of CLL cells, and T cell compartment in CLL is considered to be an important source of IL-4, our current results point to the possibility that, during CLL development, T cells acquire a unique ability to distort BTLA inhibitory function in order to maintain the longevity of malignant B cells. Therefore, we cannot exclude that the increased proliferation of CLL T cells, which is in fact an unusual feature of CLL biology, is a kind of CLL-mediated adaptive mechanism underlying the maintenance of an appropriate level of IL-4 required for B cell survival. In this regard, a substantially increasing population of CLL T cells with the sustained ability to secrete IL-4 irrespective of BTLA expression could provide effective support for leukemic B cell growth [63].

Taking into account our findings on the unimpaired level of BTLA protein expression in CLL T cells, the possibility of BTLA malfunction in T cells from our cohort of CLL patients cannot be excluded. As mentioned in the Introduction, BTLA is a unique immune checkpoint receptor with possible bidirectional signaling [rev. in 36]. Formation of the BTLA–SHP-1 complex suppresses T cell activation by inhibiting the phosphorylation of both CD28 and CD3ζ [64,65]. Conversely, when the Grb-2 protein binds to the Grb-2 motif, it recruits the p85 subunit of PI3K, triggering activation of the PI3K/AKT signaling pathway, which enhances cell activation and proliferation. This interaction also points to a potential pro-survival role of BTLA [35]. Interestingly, recent studies have revealed that BTLA expressed on antigen-presenting cells can function as a costimulatory ligand for HVEM on CD8^+^ T cells, activating the NF-κB pathway and promoting T cell proliferation [66]. Moreover, HVEM can associate with BTLA not only in a trans interaction between different cells, but also in a cis configuration when both molecules are co-expressed on the same T cell, forming BTLA–HVEM heterodimers [66]. In this cis interaction, BTLA binding inhibits HVEM-mediated costimulation, thereby blocking HVEM-dependent NF-κB transactivation, reducing T cell activation and cytokine production and maintaining T cell tolerance. Upon T cell activation, HVEM expression temporarily decreases due to internalization, while BTLA expression transiently increases, permitting trans interactions with their respective ligands. In contrast, BTLA signaling remains unaffected during cis binding with HVEM. These findings suggest a functional hierarchy in which BTLA-mediated co-inhibition predominates over HVEM-mediated co-stimulation [66,67,68]. It should be clearly emphasized that the mechanism underlying the dysfunction of BTLA or BTLA/HVEM axis in CLL T cells remains unresolved and requires confirmation in future dedicated studies. One further explanation might be that BTLA is not the only negative regulator of T cells, and various other inhibitors might influence ongoing activation.

It is also highly probable that CLL-related intrinsic factors, but not extrinsic ones (e.g., BTLA and/or HVEM), could affect proliferative activity of the CLL T cell compartment. Indeed, this finding is consistent with our recent data showing substantial expression of cyclin D2 in the T cells of CLL patients [39]. As cyclin D2 is induced in the G1 phase, its induction in CLL T cells reflects their attempt to enter the cell cycle and indicates proliferative potential [69]. Furthermore, our recent findings showing an association between genetic variants in the CCND2 gene-encoding cyclin D2 with the susceptibility and clinical course of CLL seem to strengthen the above inference [40].

At this stage, based on our functional assay, we may conclude with caution that therapeutic approaches increasing BTLA expression in T lymphocytes might contribute to undesirable changes in the microenvironment of leukemic B cells, hence promoting CLL progression, which could be an unfavorable strategy for CLL patients. This inference seems reasonable in light of our recent report showing the negative regulation of BTLA expression in B cells of CLL patients through epigenetic modulation by miR-155-5p, resulting in a lower BTLA level on CLL B cells. With the use of inhibitory siRNA targeting miR-155-5p, we demonstrate that lowering miR-155-5p levels in CLL patients partially restored the BTLA protein levels on B cells. Furthermore, since we recently found that elevated BTLA mRNA expression in CLL T cells was associated with a lack of up-regulated BTLA protein expression [33], we hypothesized that posttranslational modification may also exist in these lymphocytes. Therefore, in the present study, we performed an epigenetic modification test of BTLA expression in PBMCs from CLL by inhibition of miR-155-5p to assess its possible role in CLL pathogenesis. Previously, we showed that inhibition of miR-155-5p could partially restore BTLA expression in B cells, both in patient samples and in the MEC-1 cell line, which indirectly indicated an interaction of miR-155-5p with the BTLA gene. In the present study, we aimed to directly confirm miR155-5p-BTLA gene binding. Bioinformatic analysis revealed a canonical 7mer-A1 motif located at positions 1524–1530 (5′-GCAUUAA-3′) in the 3′UTR region of the BTLA gene, where miR-155-5p could bind. Then, using a dual-luciferase reporter assay, we demonstrated that miR-155-5p directly targets the BTLA 3′UTR and that the functional miR-155-5p MRE is located at the position suggested by bioinformatic analysis of the BTLA 3′UTR.

Our previous [25,33] and present results clearly demonstrate that BTLA mRNA is strongly overexpressed in PBMCs from CLL patients. These findings are in accordance with recent studies showing that the relative expression of BTLA in CLL cells was highest among 33 cancers compared [70]. Similar to previously published results [71], we also demonstrated a markedly higher expression of miR-155-5p in CLL patients than in healthy individuals. Furthermore, we observed that transfection with an inhibitor of miR-155 led to a marked down-regulation of miR-155 expression levels in PBMCs. Unfortunately, due to the low fraction of T cells in CLL patient PBMCs, we were not able to assess the influence of miR-155-5p inhibition on BTLA expression directly in the CLL T cell subset. Such a study and subsequent analysis require an adequate number of T cells for appropriate transfection efficiency, as well as necessary controls, which, in patients with advanced CLL, was impossible to achieve. Therefore, we cannot exclude that alterations in the miR-155-5p expression seen in CLL samples could be assigned to CLL cells rather than T cells due to their absolute numerical predominance within PBMCs. In this case, in contrast to CLL cells, T cells would display an unaffected expression of miR-155. Consistent with this suggestion, when considering the impact of miR-155-5p inhibition on BTLA protein expression in T cells, we did not observe any significant changes in the BTLA levels of all individuals studied, which strengthens our hypothesis on the unaffected level of miR-155-5p in CLL T cells. Alternatively, the true effect of miR-155-5p silencing in T cells might be partially masked by other miRNAs up-regulated in CLL, such as miR-32 [72].

## 5. Conclusions

In summary, this study indicates that immunotherapy approaches based on systemic administration of anti-miR-155-5p therapeutics would be a favorable strategy in CLL, since it does not affect BTLA expression in the T cell population and may benefit CLL patients with impaired BTLA levels on CLL cells.

## Figures and Tables

**Table 1:** Clinical and laboratory characteristics of CLL patients and healthy controls.

Parameter	CLL Patients (*n* = 20)	Healthy Controls (*n* = 15)	*p*-Value
Age (years)	67.25 ± 10.51	62.20 ± 4.93	0.0949
Gender			
Female	7	6	0.7643
Male	13	9	
Rai stage			
0	9	None	
I	6	None	
II	3	None	
III	0	None	
IV	2	None	
Binet stage			
A	15	None	
B	2	None	
C	3	None	
Blood parameters			
WBC count (1 × 10^9^/L)	73.08 ± 62.93	5.99 ± 1.41	0.000001
Lymphocyte count (1 × 10^9^/L)	66.61 ± 62.41	2.25 ± 0.65	0.000001
Hb level (g/dL)	12.69 ± 1.62	13.45 ± 0.56	0.0927
Platelet count (1 × 10^9^/L)	173.05 ± 66.52	244.93 ± 48.73	0.0006
Biochemical indicators			
LDH (U/L)	170.71 ± 61.45	148.64 ± 21.22	0.2007
β2-microglobulin (mg/L)	3.99 ± 2.21	1.38 ± 0.56	0.00003

Data are presented as means and SD or numbers. *p*-values were derived from Student’s *t*-test, the non-parametric Mann–Whitney test, or χ^2^ test (nominal values). Abbreviations: Hb, hemoglobin; LDH, lactate dehydrogenase; WBC, white blood cells.

**Table 2:** Proportion of BTLA positive and negative T cells expressing IL-4 and Ki67 in CLL patients and healthy controls.

CD3+ Cells	CLL Patients (*n* = 20)	Healthy Controls (*n* = 15)	*p*-Value
BTLA+IL-4+	39.12 ± 26.86 (5.70–78.40)	69.78 ± 26.23 (23.30–95.70)	0.0016
BTLA-IL-4+	63.61 ± 18.70 (30.30–94.10)	77.16 ± 15.27 (42.70–95.50)	0.0286
*p*-Value	0.0002	0.4265	
BTLA+Ki67+	9.28 ± 3.18 (1.00–13.80)	6.75 ± 2.82 (1.60–12.50)	0.0156
BTLA-Ki67+	7.24 ± 4.86 (0.00–18.00)	2.40 ± 1.79 (0.40–7.30)	0.0015
*p*-Value	0.1454	0.0007	

Data are presented as mean and SD (minimum–maximum). *p*-values were derived from Mann–Whitney and Student’s *t* tests.

**Table 3:** Quantitative assessment of IL-4 and Ki67 expression (MFI) in T cells of CLL patients and healthy controls.

CD3+ Cells	CLL Patients (*n* = 20)	Healthy Controls (*n* = 15)	*p*-Value
IL-4 in CD3+BTLA+	71.23 ± 52.15 (9.40–165.00)	76.95 ± 65.77 (8.70–195.50)	0.7014
IL-4 in CD3+BTLA−	29.23 ± 22.45 (8.30–59.50)	30.05 ± 21.65 (5.80–62.90)	0.8676
*p*-Value	0.00009	0.0007	
Ki67 in CD3+BTLA+	40.24 ± 18.08 (14.60–73.80)	21.59 ± 8.59 (8.90–38.30)	0.0008
Ki67 in CD3+BTLA−	7.17 ± 2.14 (0.00–10.40)	8.09 ± 3.38 (2.50–16.50)	0.6527
*p*-Value	0.00009	0.000005	

Data are presented as mean and SD (minimum–maximum). *p*-values were derived from Mann–Whitney and Student’s *t* tests.

## Data Availability

The datasets generated and/or analyzed during the current study are available from the corresponding author on reasonable request.

## Supplementary Materials

The following supporting information can be downloaded at: https://www.mdpi.com/article/10.3390/biom15111499/s1, Table S1: Clinical and laboratory characteristics of 40 CLL patients recruited for BTLA mRNA assessment in T cell population; Figure S1: Expression of IL-4 and Ki 67 in T cells from CLL patients and healthy controls (HC). a–d—gating strategy for the identification of BTLA-positive and BTLA-negative subpopulations of T cells (CD3+); e–h—representative histograms from a CLL patient demonstrating the expression of IL-4 and Ki67 in BTLA-positive or BTLA-negative T cells.

## Abbreviations

The following abbreviations are used in this manuscript:

miR	microRNA
RNA	ribonucleic acid
CLL	chronic lymphocytic leukemia
PBMC	peripheral blood mononuclear cell
siRNA	small interfering RNA
BTLA	B- and T-lymphocyte attenuator
INH	inhibitor
mRNA	messenger RNA
CD	cluster of differentiation
3′UTR	3′untranslated region
LAG-3	lymphocyte activation gene-3
TIGIT	T-cell immunoglobulin and ITIM domain
NKG2	natural killer group 2
ILT2	immunoglobulin-like transcript 2
Grb2	growth factor receptor-bound protein 2
ITIM	immunoreceptor tyrosine-based inhibitory motif
ITSM	immunoreceptor tyrosine-based switch motif
HVEM	herpesvirus entry mediator
TNFRSF	tumor necrosis factor receptor superfamily
SHP	Src homology region 2 domain-containing phosphatase
NK	natural killer
PD-1	programmed cell death protein-1
DLBCL	diffuse large B-cell lymphoma
RCC	renal cell carcinoma
BMI	body index mass
ECOG	Eastern Cooperative Oncology Group
PBS	phosphate-buffered saline
DMSO	dimethyl sulfoxide
cDNA	complementary deoxyribonucleic acid
PCR	polymerase chain reaction
WT	wild type
MUT	mutated
MFI	mean fluorescein intensity
AU	arbitrary unit
BD	Becton Dickinson
PerCP	peridinin-chlorophyll-protein
PE	phycoerythrin
FITC	fluorescein
RPMI	Roswell Park Memorial Institute
PMA	phorbol 12-myristate 23-acetate
Ion	ionomycin
BFA	brefeldin A
MoAb	monoclonal antibody
CA	California
WBC	white blood cell
Hb	hemoglobin
LDH	lactate dehydrogenase
IL	interleukin
IN	inhibitor
NC	negative control
TP53	tumor protein p53 gene
cdk	cyclin-dependent kinase
PI3K	phosphatidylinositol 3-kinase
NF-κB	nuclear factor-kappa B
