# Supplementary material for: miR-155-5p Silencing Does Not Alter BTLA Molecule Expression in CLL T Cells: Implications for Targeted Immunotherapy
Source: Biomolecules. 2025 Oct 24;15(11):1499. doi: 10.3390/biom15111499 (PMC12650147; doi:10.3390/biom15111499)
Supplement: Supplementary file 1 [file biomolecules-15-01499-s001.zip › biomolecules-3874048-supplementary.pdf]

Table S1. Clinical and laboratory characteristics of the CLL patients and healthy controls.

Parameter	CLL patients (n = 40)	Healthy Controls (n = 15)	*p*-Value
Age (years)	62.84 ± 10.51	62.20 ± 4.93	NS
Gender			
Female	19	6	NS
Male	21	9	
Rai stage			
0	11	None	
I	8	None	
II	14	None	
III	2	None	
IV	5	None	
Blood parameters			
WBC count (1 x10^9/L)	46.90 ± 30.12	5.99 ± 1.41	0.000001
Lymphocyte count (1 x10^9/L)	33.94 ± 22.81	2.25 ± 0.65	0.000001
Hb level (g/dL)	12.80 ± 1.52	13.45 ± 0.56	NS
Platelet count (1 x10^9/L)	181.60± 51.00	244.93 ± 48.73	0.0005
Biochemical indicators			
LDH (U/L)	391.10 ± 78.12	148.64 ± 21.22	0.0020
β2-microglobulin (mg/L)	3.49 ± 1.41	1.38 ± 0.56	0.00002

*Data are presented as means and SD or numbers. *P* values were derived from the Student's t-test, non-parametric the Mann–Whitney test, or χ^2 test (nominal values). Abbreviations: Hb, hemoglobin; LDH, lactate dehydrogenase; WBC, white blood cells; NS, not statistically significant

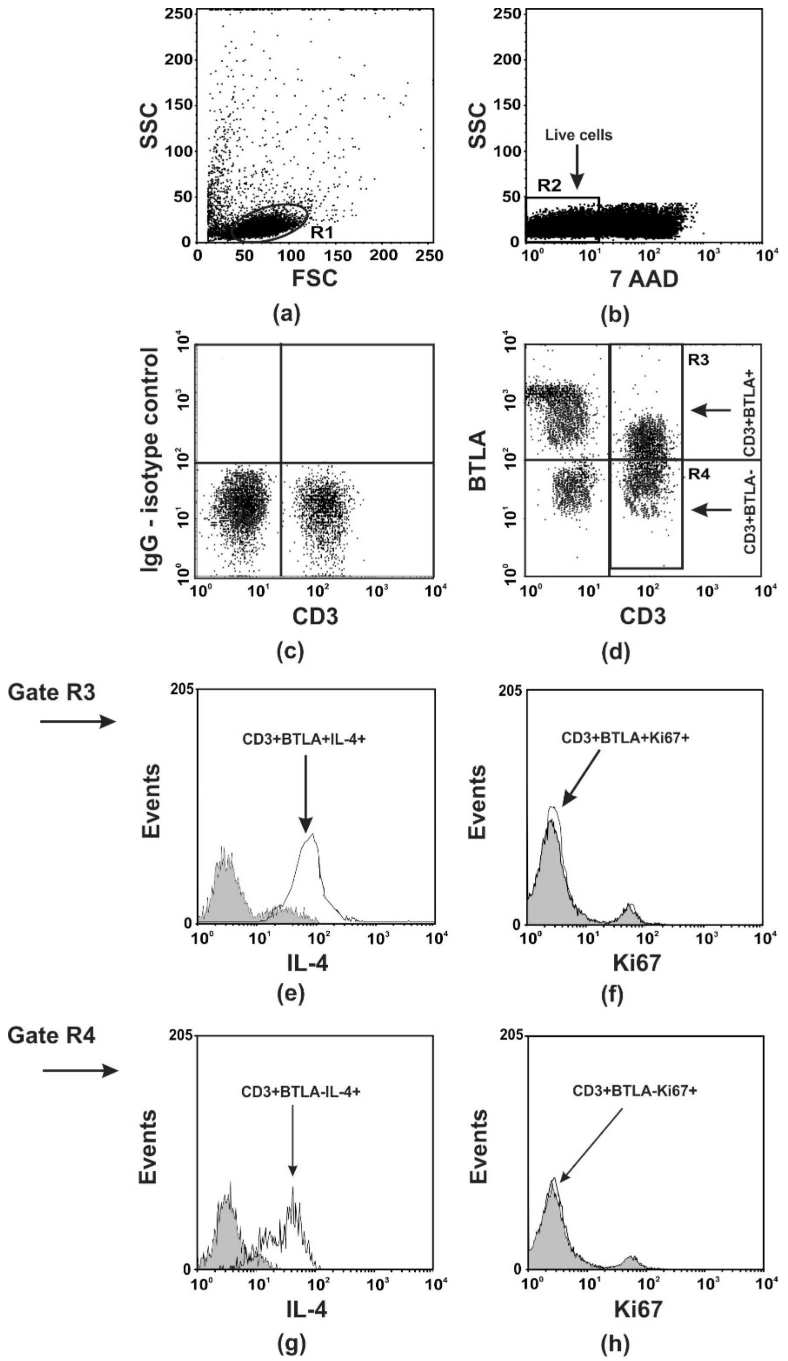

Figure S1. Gating strategy for the evaluation of proportion of BTLA positive IL-4 or Ki67 expressing cells and BTLA negative IL-4 or Ki67 expressing cells in T cell compartment in CLL patients and healthy controls. Representative plots from a CLL patient demonstrating the analytic method for the identification of BTLA-positive and BTLA-negative T cells expressing IL-4 and Ki67. (**a**) PBMCs were gated (R1) based on their FSC/SSC properties. (**b**) Gated PBMCs were then analyzed on SSC/7AAD profile for live and dead cells' discrimination; hence live cells (7-AAD-negative) were gated as region R2. (**c**–**d**) Final dot plots illustrate identification of BTLA-positive and BTLA-negative B cells. Gated live PBMCs were analyzed for CD3 and BTLA staining (dot plots: (**c**) cells stained with CD3 MoAb and isotype control IgG for BTLA (**d**) double stained cells (for CD3 and BTLA); CD3+BTLA+ cells were gated as R3, while CD3+BTLA- cells were analyzed as R4). (**e**–**h**) Final histograms show IL-4 and Ki67 fluorescence in the proportion of BTLA-positive (R3) and BTLA-negative (R4) T cells. Data were analyzed using Cell Quest software (Becton Dickinson, BD Biosciences, San Diego, CA, United States). FSC, forward scatter; SSC, side scatter; PBMC, peripheral blood mononuclear cell; MoAb, monoclonal antibody; BTLA, B and T lymphocyte attenuator; 7-AAD, (7-amino-actinomycin D) - Viability Staining Solution.
